# 1p13.2 deletion displays clinical features overlapping Noonan syndrome, likely related to *NRAS* gene haploinsufficiency

**DOI:** 10.1590/1678-4685-GMB-2016-0049

**Published:** 2016-08-04

**Authors:** Natália Duarte Linhares, Maíra Cristina Menezes Freire, Raony Guimarães Corrêa do Carmo Lisboa Cardenas, Heloisa Barbosa Pena, Katherine Lachlan, Bruno Dallapiccola, Carlos Bacino, Bruno Delobel, Paul James, Ann-Charlotte Thuresson, Göran Annerén, Sérgio D. J. Pena

**Affiliations:** 1 Laboratório de Genômica Clínica, Faculdade de Medicina, Universidade Federal de Minas Gerais (UFMG), Belo Horizonte, MG, Brazil; 2 Departamento de Bioquímica e Imunologia, Instituto de Ciências Biológicas, Universidade Federal de Minas Gerais (UFMG), Belo Horizonte, MG, Brazil; 3 Laboratório Gene – Núcleo de Genética Médica, Belo Horizonte, MG, Brazil; 4 Wessex Clinical Genetics Service, Princess Anne Hospital, Southampton, United Kingdom; 5 Bambino Gesù Children Hospital, Rome, Italy; 6 Department of Molecular and Human Genetics, Baylor College of Medicine, Houston, TX, USA; 7 Centre de Génétique Chromosomique, GH de l'Institut Catholique de Lille - Hopital Saint Vincent de Paul, Lille, France; 8 Victorian Clinical Genetics Service, Melbourne, Victoria, Australia; 9 Department of Immunology, Genetics and Pathology, The Rudbeck Laboratory, Uppsala University, Uppsala, Sweden

**Keywords:** 1p13.2 deletion, Noonan syndrome type 6, *NRAS* gene, RASopathy, unmasking heterozygosity

## Abstract

Deletion-induced hemizygosity may unmask deleterious autosomal recessive variants and be a cause of the phenotypic variability observed in microdeletion syndromes. We performed complete exome sequencing (WES) analysis to examine this possibility in a patient with 1p13.2 microdeletion. Since the patient displayed clinical features suggestive of Noonan Syndrome (NS), we also used WES to rule out the presence of pathogenic variants in any of the genes associated with the different types of NS. We concluded that the clinical findings could be attributed solely to the 1p13.2 haploinsufficiency. Retrospective analysis of other nine reported patients with 1p13.2 microdeletions showed that six of them also presented some characteristics of NS. In all these cases, the deleted segment included the *NRAS* gene. Gain-of-function mutations of *NRAS* gene are causally related to NS type 6. Thus, it is conceivable that *NRAS* haploinsufficiency and gain-of-function mutations may have similar clinical consequences. The same phenomenon has been described for two other genes belonging to the Ras/MAPK pathway: *MAP2K2* and *SHOC2*. In conclusion, we here report genotype-phenotype correlations in patients with chromosome 1p13.2 microdeletions and we propose that *NRAS* may be a critical gene for the NS characteristics in the patients.

## Introduction

Patients with chromosomal deletions may present with variable clinical phenotypes for different reasons. First, the deletions frequently vary in size and the phenotypic alterations may depend on which loci are deleted in the specific case, as exemplified by the contiguous gene syndromes such as, for instance, Langer-Giedion syndrome (OMIM #150230) and Williams-Beuren syndrome (OMIM #194050). Second, the phenotypic effect of the deleted segment may be modulated by some specific regions of the rest of the genome, in analogy with autosomal dominant diseases with variable expressivity and as, for instance, is the case in digenic inheritance (Schaffer, 2013). Third, early postzygotic events, and/or environmental factors may influence phenotypic discordances, such as in cases of monozygotic twins with discordant phenotype and chromosome 22q11.2 microdeletion (Yamagishi *et al*., 1998). Fourth, the clinical phenotype may be influenced by the presence of a pathogenic variant in a gene located in the region homologous to the deleted segment, in the intact member of the chromosome pair. This phenomenon has been termed "unmasking heterozygosity" (UH) and when it occurs the phenotype of a recessive disease might emerge from the microdeletion due to hemizygosity (Coman and Gardner, 2007; Poot, 2012). To our knowledge, the earliest example of this phenomenon was described in retinoblastoma (OMIM #180200), which may occur because a chromosomal deletion may cause hemizygosity for an *RB1* (OMIM *614041) pathogenic variant on chromosome 13q14.

Next generation sequencing (NGS) techniques, such as whole genome sequencing (WGS) and whole exome sequencing (WES) allow the screening of the genome including the non-deleted allele for variants that may contribute to variable phenotypic expression in deletion syndromes. Accordingly, NGS has been reported to reveal deleterious variants unmasked by hemizygous deletions in individuals with 22q11.2 deletion syndrome (OMIM #192430; #188400) (McDonald-McGinn *et al*., 2013), with mental retardation or multiple congenital abnormalities and hemizygous deletions (Hochstenbach *et al*., 2012), and with thrombocytopenia absent radius syndrome (OMIM #274000) (Albers *et al*., 2012).

Since UH can be revealed using exome sequencing, we used it in a genotype-phenotype correlation study of chromosome 1p13.2 microdeletions. We here report the case of a 21-year-old man with a multisystem phenotype containing some features of Noonan syndrome (OMIM #613224) and 1p13.2 deletion extending from 1:112,096,417 to 1:115,805,157 (hg19) diagnosed by aCGH. Exome sequencing analysis examined the possibility of a recessive pathogenic variant unmasked by his hemizygous deletion and also verified if he had pathogenic variants in any of the Noonan Syndrome genes. We concluded that the clinical characteristics of the patient could be attributed to the 1p13.2 haploinsufficiency. Additionally, we compared his clinical characteristics with the phenotype of six patients with similar deletions overlapping the one of our patient reported previously in DECIPHER database and three individuals reported in the literature (Mattia *et al*., 1992; Bisgaard *et al*., 2007; Fitzgibbon *et al*., 2009). On these bases we propose that haploinsufficiency of *NRAS* gene (OMIM *164790) may be important in determining the clinical phenotype of this microdeletion.

### Clinical report

Our patient was a 21-year-old man born of healthy unrelated parents (DECIPHER patient no. 274660). He was born at 37 weeks of pregnancy by cesarean delivery for breech presentation. Birth weight was 2,720 g (3rd-10th centile), with length of 47 cm (10th centile) and occipital frontal circumference (OFC) 34.5 cm (25-50th centile). He presented with hypotonia and feeding difficulties in infancy. His motor development was delayed, having walked independently at 22 months of age. At 3 years-old he spoke single words and at 4 years-old he spoke sentences. He never learned to write or to read. Since infancy he has had recurring bouts of vomiting. Colonoscopy revealed a polypoid mucosa in the terminal ileum and chronic enteritis. Ophthalmologic exams showed myopia, with normal fundus. Echocardiography and nuclear magnetic resonance of the brain were normal.

**Figure 1:**
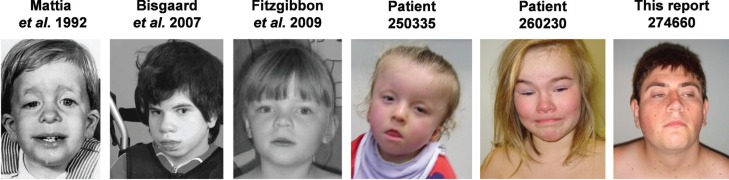
Frontal view of patients with deleted *NRAS*. Patients present features of Noonan Syndrome 6, including macrocephaly, short/webbed neck, low hairline, skin abnormalities, triangular face with age, low-set ears, arched eyebrows, hypertelorism, ptosis, downslating palpebral fissures and epicanthal folds. Considering the patients with *NRAS* deletion, we did not have a picture of patient 253793 and 258063.

**Figure 2:**
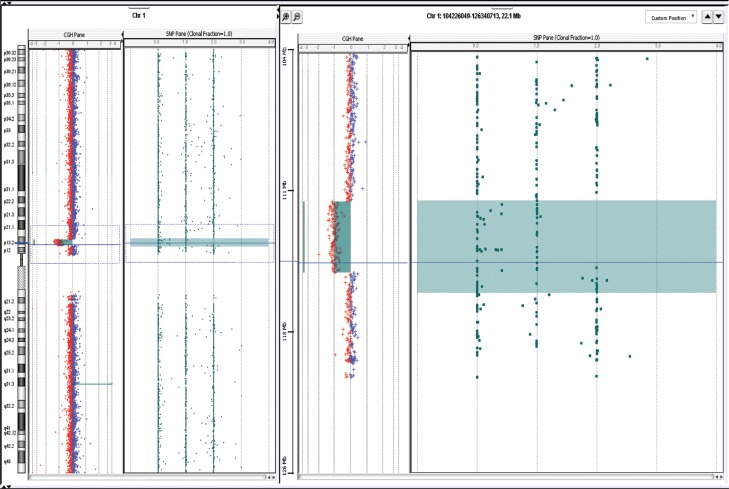
Copy number profile of chromosome 1 of our patient obtained by aCGH. The chromosome 1 copy number imbalances are indicated on the left panel and shown in detail on the right panel: the alterations marked by the square show a ~3.71 Mb 1p13.2 deletion.

He was evaluated by a geneticist at 20 years of age, when his weight was 68.5 kg (25-50th centile), height 161 cm (< 3rd percentile) and OFC 53 cm (< 3rd percentile). Besides the short stature, his phenotype had several features reminiscent of Noonan Syndrome, including intellectual disability, ptosis, low hairline at the nape, broad neck, excess of pigmented nevi resembling lentigines, *pectus excavatum* and scoliosis (see Figure 1). Complementary examinations showed patellar chondromalacia, osteoporosis, asymmetry of the lower limbs, nephrolithiasis with increased loss of K^+^ and Ca^+^ in urine, hypercholesterolemia and primary hypothyroidism. The conventional karyotype of the patient and his parents were normal. However, subsequent aCGH analysis (Agilent SurePrint G3 4x180K) revealed a 3.71 Mb microdeletion on chromosome 1p13.2 (chr1:112,096,417–115,805,157; UCSC Genome Browser hg19) (Figure 2).

## Material and Methods

### Samples and DNA isolation

This study was approved by the Research Ethics Committee of Universidade Federal de Minas Gerais and by the Brazilian National Committee of Ethics in Research (CONEP – Brazil) with number 854.709. Written informed consent including permission for publication was provided by the parents. Genomic DNA was isolated from peripheral blood of the patient using a modified salting out procedure (Miller *et al*., 1988).

### Whole exome sequencing and analysis

Whole exome sequencing was performed in the patient sample by The Centre for Applied Genomics, The Hospital for Sick Children (Toronto, Canada) using the Agilent SureSelect Human All Exon V4 Kit (Agilent Technologies) and SOLiD 5500xl platform (Applied Biosystems). All data were aligned to the hg19/GRCh37 reference genome build via BFAST and BWA aligner. Variants were quality trimmed using the Genome Analysis Toolkit (GATK 1.1.28) and they were annotated for functional effect by SnpEff 2.0.5. SnpEff also provided a simple assessment of the putative impact of the variant (e.g. High, Moderate, or Low impact). Alignment, calling and annotation of the variants against databases such as 1000 Genomes (April 2012 release), NHLBI Exome Sequencing Project (ESP6500) and Single Nucleotide Polymorphism database (dbSNP137) were done using a software developed in-house called *Mendel,MD* (Cardenas *et al*., 2015). The prediction software SIFT (Sorting Intolerant From Tolerant) was used in order to estimate whether a given amino acid substitution affected protein function. SIFT prediction is based on the degree of conservation of amino acid residues in sequence alignments derived from closely related sequences and on the physical properties of amino acids.

## Results

Whole exome sequencing of the patient sample identified 76,822 variants. Approximately 26,863 of these variants passed in the GATK quality filters and had a Minor Allele Frequency (MAF) ≤ 0.01 in the 1000 Genomes, ESP6500 and dbSNP137. In the deleted region, which extended from 1:112,096,417 to 1:115,805,157 (hg19), exome analysis revealed 35 hemizygous variants that passed in GATK quality filters. Only 10 of these variants were considered to have moderate or high impact by SnpEff, which means that they are assumed to have high disruptive impact in the protein or might change protein effectiveness (examples: stop gained, frameshift, missense and splice donor variants). Based on the assumption that the potential damaging variants are rare, the variants were then filtered considering an allele frequency < 0.01 in the 1000 Genomes database, ESP6500 and dbSNP137, however no plausibly pathogenic variant was retained. Besides, all of these 10 variants were predicted as tolerated by SIFT (score ≥ 0.05) (Table S1). In addition, we verified by exome analysis that the patient did not have any pathogenic variants in the genes associated with Noonan Syndrome and other rasopathies (*PTPN11*, *SHOC2*, *KRAS*, *CBL*, *SOS1*, *RAF1*, *HRAS*, *NRAS*, *RASA1*, *SPRED1*, *BRAF*, *MAP2K1*, *MAP2K2, RIT1* and *RRAS*) (Rauen, 2013; Ekvall *et al*., 2015).

The deleted 1p13.2 region of our patient contained 69 genes according to the NCBI Map Viewer Annotation Release 105, of which 28 were listed in OMIM (Table S2). However, only eight of these genes were known to be associated with Mendelian disease phenotypes (Table S3). Among the recessive disease genes there was the *TSHB* gene (OMIM *188540), in which homozygous mutations had been reported in patients with congenital nongoitrous hypothyroidism-4 (OMIM #275100). Our patient had hypothyroidism, but it was not congenital.

In order to perform a genotype-phenotype correlation study of chromosome 1p13.2 microdeletions, we compared the clinical characteristics of our patient to those of previously described cases (Table 1 and Figure 1). Isolated chromosome 1p13.2 microdeletions are not common; we could find only three individuals reported in the literature (Mattia *et al*., 1992; Bisgaard *et al*., 2007; Fitzgibbon *et al*., 2009). Additionally, six patients with isolated deletions partially overlapping the one of our patient had been reported previously in DECIPHER database. All these nine patients were included in our analysis.

**Table 1:** Clinical features of 10 patients with isolated 1p13.2 microdeletions.

	Mattia *et al*. 1992	Bisgaard *et al*. 2007	Fitzgibbon *et al*. 2009	Patient 250335	Patient 253793	Patient 256753	Patient 257066	Patient 258063	Patient 260230	This report 274660
Extension^a^	ND	~103804508-115747737	~114609456-116035987	105976094-120529725	113268038-117119460	102041407-112285318	95286548-113219619	113525828-115713872	115018964-118929584	112096417-115805157
Size (Mb)	ND	11.9-14	1.4-3.1	14.55	3.85	10.24	17.93	2.19	3.91	3.71
*NRAS* deleted	+	+	+	+	+	-	-	+	+	+
Age at last examination	2 years	13 years	5 years	3 years	38 years	4 years	2 years	8 years	8 years	21 years
Gender	Male	Female	Female	Female	Male	Male	Female	Female	Female	Male
Infantile feeding difficulties	ND	ND	+	No	No	+	+	No	No	+
Facial features of Noonan Syndrome 6^b^	+	+	+	No^c^	+^d^	No	No	No^c^	No^c^	+
Short stature	No	+	ND	+	+	No	+	No	+	+
Macrocephaly	No	No	ND	Relative	+	No	Relative	+	+	No
Webbed neck	Short neck	+	ND	Short neck	Short neck	No	Short and broad neck	No	No	Broad neck
Ophthalmological abnormalities	No	Iris coloboma	ND	Iris coloboma	No	No	Papillary coloboma	Astigmatism	Strabismus	Myopia
Motor delay/muscular hypotonia	+	+	+	+	+	+	+	+	+	+
Intellectual disability	NA	+	+	+	+	+	NA	+	+	+
Speech delay	+	+	+	+	+	+	+	+	+	+
Low hairline	ND	+	ND	+	No	No	ND	No	No	+
Skin abnormalities	Forehead *nevus flammeus*	ND	ND	No	Several lentigenes	No	No	No	Several vitiligo, dry skin	Several lentigenes
Congenital heart defects	No	ND	ND	+^e^	No	No	ND	No	No	No

NA, not applicable; ND, not determined.

a UCSC Genome Browser hg19 coordinates (except for Bisgaard *et al*., 2007). Mattia *et al*. (1992) did not perform molecular analysis.

b Facial features of Noonan Syndrome 6 include: triangular face with age, low-set ears, hypertelorism, palpebral ptosis, downslating papebral fissures, epicanthal folds.

c Overall gestalt is not strongly reminiscent of Noonan syndrome.

d Patient 253793 is an adult, and facial features of Noonan syndrome are less recognizable in adult individuals.

e Ventricular and atrial septum defect.

**Figure 3:**
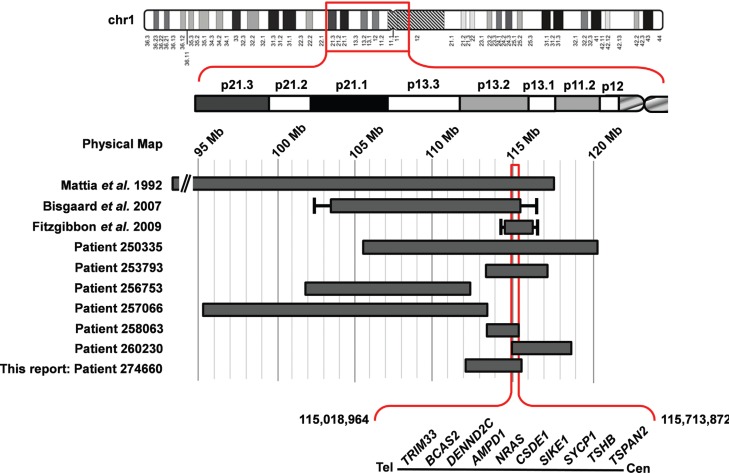
Schematic representation of the deleted segments in our patient and those previously reported with isolated 1p13.2 microdeletions. Except for the patient described by Mattia *et al*. (1992), who has a microdeletion from 1p13 to 1p22.3, all other patients have their breakpoints defined by molecular methods. Ideogram of chromosome 1, physical map and deleted segments are indicated according to their placement on the Ensembl Genome Browser.

**Figure 4:**
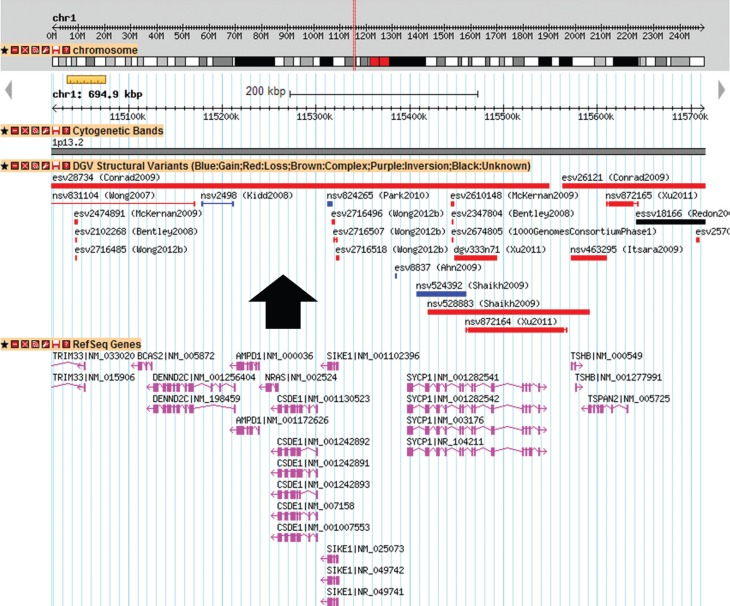
Genomic variants listed in the Database of Genomic Variants (DGVbeta) in the smallest region of overlap between the patients (chr1:115,018,964–115,713,872) (hg19). The black arrow indicate the region less populated with CNVs.

The deletions in the several patients ranged from 2.19 Mb to 17.93 Mb in size and eight of them included a region extending from 115,018,964 to 115,713,872 (hg19). The region of overlap of all patients contained 10 genes: *TRIM33*, *BCAS2*, *DENND2C*, *AMPD1*, *NRAS*, *CSDE1*, *SIKE1*, *SYCP1*, *TSHB*, *TSPAN2* (Figure 3). The Database of Genomic Variants (DGVbeta) lists numerous copy-number variations (CNVs) in individuals with normal phenotypes within this region. The segment with the least number of CNVs was the one containing the genes *AMPD1*, *NRAS* and *CSDE1* (Figure 4).

The patient reported by Fitzgibbon *et al*. (2009) presented congenital insensitivity to pain and temperature, which led the authors to propose that this might be a consequence of haploinsufficiency of the *NGF* gene (OMIM *162030), which is related to hereditary sensory and autonomic neuropathy type V, an autosomal recessive disease (sequencing of the undeleted homologous chromosome had not shown any pathogenic variants). However, in disagreement with this suggestion, *NGF* was also deleted in three patients described in DECIPHER, none of whom presented congenital insensitivity to pain and temperature (patients no. 250335, 253793 and 260230). In relation to coloboma, Bisgaard *et al*. (2007) hypothesized that haploinsufficiency of the *WNT2B* gene (OMIM *601968) might have caused it in their patient. Indeed, this gene was deleted in two other patients with ocular coloboma (Patients no. 250335 and 257066), but it was also deleted in our patient, who did not have this malformation.

## Discussion

We here report a patient with isolated 1p13.2 microdeletion diagnosed by aCGH analysis. We performed exome sequencing in the patient in order to check for possible unmasking heterozygosity and to exclude variants in any of the genes related to Noonan Syndrome or other RASopathies. The analysis of complete exome sequencing (WES) identified 35 hemizygous variants in the deleted region. However, evaluation of frequency in the population and bioinformatics protein predictions did not indicate pathogenicity of these variants. Moreover, we did not find pathogenic variants in any of the genes known to be associated with Noonan Syndrome. Consequently, we conclude that the phenotype of our patient is exclusively related to his hemizygous 1p13.2 state.

We then tried to identify in the deletion region, genes possibly related with the phenotype of our patient and others. Our attention was drawn to the presence of deletion of *NRAS* for four reasons:

1. The phenotype of our patient and also of other patients with the 1p13.2 deletion includes several features of Noonan Syndrome (Table 1 and Figure 1);
2. Variants of *NRAS* have been described as causing Noonan Syndrome, Type 6 (OMIM #613224), an autosomal dominant disorder (Cirstea *et al*., 2010). However, these have been shown to be gain-of-function mutations;
3. Haploinsufficiency Index predictions (HI) shown in the DECIPHER database for all the genes deleted in our patient, revealed that the *NRAS* gene was the one with higher rank of HI (which suggested that this gene is more likely to exhibit haploinsufficiency) (Huang *et al*., 2010);
4. The region surrounding *NRAS* is rarely seen in polymorphic CNVs (Figure 4).

*NRAS* is a member of the Ras subfamily, which includes 36 members; *KRAS*, *HRAS* and *NRAS* have been the most studied because of their critical roles in human oncogenesis (Wennerberg *et al*., 2005). Ras proteins belong to a class of signal-transducing GTPases, which cycle between an active GTP-bound and an inactive GDP-bound conformation. Alterations of conditions, including gene variants, may result in stabilization of Ras proteins in their active state leading to malignant transformation (Scheffzek *et al*., 1997). Ras proteins activate several pathways, including the Ras/mitogen activated protein kinase (MAPK) pathway (Wennerberg *et al*., 2005). This pathway plays an essential role in regulating the cell survival, differentiation, proliferation and apoptosis, among many others (Pearson *et al*., 2001).

Germline variants in genes that encode components or regulators of the Ras/MAPK pathway have been shown to cause developmental syndromes collectively referred to as RASopathies. These disorders include several forms of Noonan syndrome, including Costello syndrome (OMIM #218040), cardio-facio-cutaneous syndrome (OMIM #115150), and capillary malformation–arteriovenous malformation syndrome (OMIM #608354) (Rauen, 2013). In all of these cases the mutations have been shown to involve gain-of-function, increasing activity in the Ras/MAPK pathway. It is believed that this might be the reason why they are clinically similar, with overlapping phenotypic features including characteristic facial features, cardiac defects, cutaneous abnormalities, neurocognitive delay and an increased cancer risk (Tidyman and Rauen, 2009; Rauen, 2013).

Few heterozygous mutations have been described as cause of the Noonan type 6 (NS6) and they have all been associated with gain-of-function in *NRAS* (De Filippi *et al*., 2009; Cirstea *et al*., 2010; Runtuwene *et al*., 2011; Denayer *et al*., 2012; Kraoua *et al*., 2012; Ekvall *et al*., 2015). Cirstea *et al*. (2010) expressed each mutant as yellow fluorescent protein–NRAS fusion proteins in cells from the COS-7 line and showed for example that NRAS substitutions p.Thr50Ile or p.Gly60Glu resulted in enhanced phosphorylation of MEK and ERK in the presence of serum or after epidermal growth factor (EGF) stimulation. In addition, the authors observed that the p.Gly60Glu NRAS mutant accumulated constitutively in the active, GTP-bound form (Cirstea *et al*., 2010). Runtuwene *et al*. (2011) showed that similar effects occurs in GTP-bound in p.Ile24Asn mutants and they revealed that p.Gly60Glu, p.Ile24Asn and the positive control (p.Gly12Val) mutants also enhanced MAPK activation. These patients with *NRAS* mutations often show a relatively mild phenotype of typical Noonan features, including hypertelorism, low-set ears, short stature, webbed neck, low hairline, thorax deformities, motor delay/muscular hypotonia and lentigenes/cafe-au-lait spots (reviewed by Kraoua *et al*., 2012; Ekvall *et al*., 2015).

On the other hand, our patient and other cases with pure chromosome 1p13.2 microdeletions and hemizygosity for *NRAS* reviewed by us from the literature or from the DECIPHER database all seemed to present several clinical features of Noonan Syndrome (Table 1 and Figure 1). The presence of such features related to Noonan Syndrome in patients with deletion of *NRAS* and presumably haploinsufficiency of the gene product appears *prima facie* paradoxical.

We searched the literature and we have found three similar examples. In Noonan syndrome, Edwards *et al*. (2014) described a 5-year-old male with two *de novo* pathogenic *PTPN11* variants in *cis* (OMIM *176876). The double-mutant gene product (SHP-2) was found to be catalytically impaired. More recently, Chen *et al*. (2014) described a patient with a Noonan-like phenotype associated with a deletion of the 10q25.2 chromosomal region that included the *SHOC2* gene (OMIM *602775). Gain-of-function mutations in this gene have been shown to cause a Noonan Syndrome-like syndrome. In the same vein, Nowaczyk *et al*. (2014) reported seven patients with deletions of chromosome 19p13.3 including the *MAP2K2* gene (OMIM *601263) and phenotype features of Cardio-facio-cutaneous syndrome, which is a RASopathy known to be caused by activating mutations of the *BRAF*, *MAP2K1*, *MAP2K2*, or *KRAS* genes. On these bases, these authors proposed that haploinsufficiency of *MAP2K2/MEK2* "appears to be a new model of a RASopathy, where a deletion of one of the components of the pathway, MEK2, results in a RASopathy-like phenotype" (Nowaczyk *et al*., 2014). We believe that the association here described of 1p13.2 microdeletion involving hemizygosity for *NRAS* and clinical features of Noonan syndrome, represent a further example of the model proposed by Nowaczyk *et al*. (2014), according to which, haploinsufficiency of a gene in the Ras/MAPK pathway may cause dysregulation of the pathway and produce a RASopathy phenotype.

## Supplementary material

The following online material is available for this article:

Table S1 - Hemizygous variants within the deleted region that passed in GATK quality filters.

Table S2 - Genes deleted in the patient according to the NCBI Map Viewer Annotation Release 105.

Table S3 - Deleted genes in our patient associated with Mendelian disease phenotypes in OMIM.
