# Supplementary material for: 1p13.2 deletion displays clinical features overlapping Noonan syndrome, likely related to NRAS gene haploinsufficiency
Source: Genet Mol Biol. 2016 Aug 4;39(3):349–57. doi: 10.1590/1678-4685-GMB-2016-0049 (PMC5004838; doi:10.1590/1678-4685-GMB-2016-0049)
Supplement: Supplementary file 1 [file 1415-4757-gmb-1678-4685-GMB-2016-0049-Suppl01.pdf]

Table S1 - Hemizygous variants within the deleted region that passed in GATK quality filters.

Pos	Variant_ID	Ref	Alt	Qual	Read depth	Gene	Genomes1k MAF	dbSNP MAF	ESP MAF	SIFT	Impact	Func_class	Consequence
115231459	rs386500536	C	T	516.14	16	AMPD1	0.000998403	0.002296					intron_variant
114438951	rs1217401	A	G	1548.19	44	AP4B1	0.376997	0.3466	0.437952	0.07	MODERATE	MISSENSE	missense_variant
114424601	rs1217381	C	T	2843.61	78	BCL2L15	0.999401	0.9986	0.000154	0.63	MODERATE	MISSENSE	missense_variant
114423653	rs1217382	C	T	793.61	29	BCL2L15	0.401158	0.3691	0.456198				3_prime_UTR_variant
115128087	rs386561521	A	G	377.98	11	DENND2C	0.828275	0.7989					3_prime_UTR_variant
115144703	rs386480540	A	G	1210.74	33	DENND2C	0.998403	0.9972	0.007535				intron_variant
115168097	rs12136548	T	C	1069.38	29	DENND2C	0.125599	0.163	0.221821	0.73	MODERATE	MISSENSE	missense_variant
112269872	rs197434	G	A	3156.61	90	FAM212B	0.860623	0.8476	0.14255		LOW	SILENT	synonymous_variant
113657092	rs1216793	A	C	1495.13	40	LRIG2	0.901757	0.9118	0.096878		LOW	SILENT	synonymous_variant
113658887	rs1216791	G	A	962.27	28	LRIG2	0.789936	0.7934	0.23543				intron_variant
114191994	rs1743594	G	A	817.72	23	MAGI3	0.338658	0.3242	0.410503				intron_variant
114092306	rs10858004	A	G	1179.21	31	MAGI3	0.16254	0.1543					intron_variant
114161116	rs1217200	C	A	122.01	5	MAGI3	0.671925	0.6396					intron_variant
114267347	rs1230640	C	T	1441.05	40	PHTF1	0.703474	0.6662	0.194679				intron_variant
114240319	rs3835263	T	TA	1107.46	29	PHTF1	0.678115		0.214936				3_prime_UTR_variant
114249382	rs6698586	C	T	2900.02	78	PHTF1	0.679313	0.6488	0.221086				intron_variant
114281438	.	C	A	104.77	8	PHTF1							intron_variant
113254648	rs78366259	A	G	987.75	28	PPM1J	0.0109824	0.01286	0.021298	0.19	MODERATE	MISSENSE	missense_variant
113255389	rs386481312	C	T	804.48	22	PPM1J	0.967851	0.9706	0.039136	1.0	MODERATE	MISSENSE	missense_variant
114377568	rs2476601	A	G	1492.69	45	PTPN22	0.972644	0.9578	0.068353	1.0	MODERATE	MISSENSE	missense_variant
114377093	rs386573918	A	G	797.01	22	PTPN22	0.701278	0.663	0.197324				intron_variant
112106444	rs1591712	G	A	3174.29	99	RAP1A	0.894768	0.9132	0.105172				intron_variant
112251983	rs3738300	C	G	3001.04	78	RAP1A	0.322484	0.3251					intron_variant
115316998	.	A	G	186.53	9	SIKE1							splice_region_variant
113456546	rs1049434	A	T	4655.27	122	SLC16A1	0.676717	0.6575	0.333769	1.0	MODERATE	MISSENSE	missense_variant

Pos	Variant_ID	Ref	Alt	Qual	Read depth	Gene	Genomes1k MAF	dbSNP MAF	ESP MAF	SIFT	Impact	Func_class	Consequence
113098534	rs6658555	C	T	1723.01	48	ST7L	0.1248	0.1593	0.204444	0.07	MODERATE	MISSENSE	missense_variant
113083439	rs10745330	C	T	635.67	19	ST7L	0.489816	0.5303					intron_variant
115398044	rs60659641	GC	G	1068.49	0	SYCP1	0.9998	0.9844	0.007352				intron_variant
114680540	rs17032441	C	T	3158.91	85	SYT6	0.207668	0.2112	0.203983		LOW	SILENT	synonymous_variant
114948281	rs6537825	A	G	4024.46	106	TRIM33	0.85643	0.8384	0.057666	1.0	MODERATE	MISSENSE	missense_variant
114940632	rs5777192	CA	C	241.46	3	TRIM33							intron_variant
115576023	rs386515121	A	G	947.38	27	TSHB	0.983626	0.9853	0.024758	0.58	MODERATE	MISSENSE	missense_variant
115631924	rs4240540	C	T	136.87	5	TSPAN2	0.973043	0.95					intron_variant
115601615	rs7523818	A	G	1152.42	30	TSPAN2	0.965655	0.955	0.065893				intron_variant
113063125	rs910697	A	G	4607.74	119	WNT2B	0.483427	0.5239	0.47578		LOW	SILENT	synonymous_variant
