# Supplementary material for: 1p13.2 deletion displays clinical features overlapping Noonan syndrome, likely related to NRAS gene haploinsufficiency
Source: Genet Mol Biol. 2016 Aug 4;39(3):349–57. doi: 10.1590/1678-4685-GMB-2016-0049 (PMC5004838; doi:10.1590/1678-4685-GMB-2016-0049)
Supplement: Supplementary file 2 [file 1415-4757-gmb-1678-4685-GMB-2016-0049-Suppl02.pdf]

Table S2 - Genes deleted in the patient according to the NCBI Map Viewer Annotation Release 105.

start	stop	Symbol	O	Link	Cyto	Description
112025970	112106602	ADORA3	-	OMIM	1p13.2	adenosine A3 receptor
112141629	112150946	RP5-836N10.1	-		1p13.2	uncharacterized LOC100129269
112162405	112256807	RAP1A	+	OMIM	1p13.3	RAP1A, member of RAS oncogene family
112190850	112192219	KRT18P57	-		1p13.2	keratin 18 pseudogene 57
112264686	112298419	FAM212B	-		1p13.2	family with sequence similarity 212, member B
112282463	112290420	FAM212B-AS1	+			FAM212B antisense RNA 1
112287939	112298158	LOC101928718	-			uncharacterized LOC101928718
112298190	112310199	DDX20	+	OMIM	1p21.1-p13.2	DEAD (Asp-Glu-Ala-Asp) box polypeptide 20
112318444	112532147	KCND3	-	OMIM	1p13.3	potassium voltage-gated channel, Shal-related subfamily, member 3
112532593	112541464	LOC643355	+		1p13.2	uncharacterized LOC643355
112715822	112720236	LOC101928754	-			uncharacterized LOC101928754
112905821	112906283	LOC171419	+		1q12	thioredoxin pseudogene
112938800	113003786	CTTNBP2NL	+	OMIM	1p13.2	CTTNBP2 N-terminal like
113004392	113004455	MIR4256	-			microRNA 4256
113010040	113063910	WNT2B	+	OMIM	1p13	wingless-type MMTV integration site family, member 2B
113066140	113162040	ST7L	-		1p13.2	suppression of tumorigenicity 7 like
113162075	113214241	CAPZA1	+	OMIM	1p13.2	capping protein (actin filament) muscle Z-line, alpha 1
113168494	113168847	MRPL53P1	-		1p13.2	mitochondrial ribosomal protein L53 pseudogene 1
113177351	113177403	RNU7-70P	-		1p13.2	RNA, U7 small nuclear 70 pseudogene
113217048	113243368	MOV10	+	OMIM	1p13.2	Mov10, Moloney leukemia virus 10, homolog (mouse)
113243749	113250025	RHOC	-	OMIM	1p13.1	ras homolog family member C

start	stop	Symbol	O	Link	Cyto	Description
113252616	113257950	PPM1J	-	OMIM	1p13.2	protein phosphatase, Mg2+/Mn2+ dependent, 1J
113261619	113269857	FAM19A3	+		1p13.2	family with sequence similarity 19 (chemokine (C-C motif)-like), member A3
113290717	113291097	LOC128322	+		1p13.2	nuclear transport factor 2-like
113362791	113393265	RP11-426L16.8	-		1p13.2	uncharacterized LOC100996702
113433214	113433593	RPL39P8	-		1p13.2	ribosomal protein L39 pseudogene 8
113454469	113498975	SLC16A1	-	OMIM	1p12	solute carrier family 16 (monocarboxylate transporter), member 1
113465972	113467295	AKR7A2P1	+		1p12	aldo-keto reductase family 7, member A2 pseudogene 1
113499037	113506694	SLC16A1-AS1	+		1p13.2	SLC16A1 antisense RNA 1
113554309	113615724	RP11-31F15.2	-			uncharacterized LOC100996251
113615792	113667824	LRIG2	+	OMIM	1p13.1	leucine-rich repeats and immunoglobulin-like domains 2
113668114	113669577	RLIMP2	-			ring finger protein, LIM domain interacting pseudogene 2
113711611	113712051	RPS19P2	+		1p13.2	ribosomal protein S19 pseudogene 2
113739403	113748875	LOC643441	-		1p13.2	uncharacterized LOC643441
113741429	113741913	RPS15P1	+		1p13.2	ribosomal protein S15 pseudogene 1
113933087	114228545	MAGI3	+		1p12-p11.2	membrane associated guanylate kinase, WW and PDZ domain containing 3
113992328	113993307	LOC100421402	+			heterogeneous nuclear ribonucleoprotein A1 pseudogene
114119373	114119516	MTND5P20	-			MT-ND5 pseudogene 20
114239824	114302165	PHTF1	-	OMIM	1p13	putative homeodomain transcription factor 1
114241506	114242228	RPS2P14	-		1p13	ribosomal protein S2 pseudogene 14
114304454	114355070	RSBN1	-		1p13.2	round spermatid basic protein 1
114354977	114361461	RP5-1073O3.2	+			uncharacterized LOC101928822

start	stop	Symbol	O	Link	Cyto	Description
114356433	114414375	PTPN22	-	OMIM	1p13.2	protein tyrosine phosphatase, non-receptor type 22 (lymphoid)
114399257	114443859	AP4B1-AS1	+		1p13.2	AP4B1 antisense RNA 1
114419436	114430230	BCL2L15	-		1p13.2	BCL2-like 15
114437370	114447741	AP4B1	-	OMIM	1p13.2	adaptor-related protein complex 4, beta 1 subunit
114447915	114456708	DCLRE1B	+	OMIM	1p13.2	DNA cross-link repair 1B
114466623	114471880	RP5-1073O3.7	-			uncharacterized LOC101928846
114471819	114520491	HIPK1	+	OMIM	1p13.2	homeodomain interacting protein kinase 1
114522030	114524875	OLFML3	+	OMIM	1p13.2	olfactomedin-like 3
114544055	114545965	RPL13AP10	-		1p13.2	ribosomal protein L13a pseudogene 10
114631914	114696472	SYT6	-	OMIM	1p13.2	OTTHUMP00000195668
114821624	114821908	MRP63P1	-		1p13.1	mitochondrial ribosomal protein 63 pseudogene 1
114896608	114897781	LOC100421116	+			trafficking protein, kinesin binding 2 pseudogene
114935399	115053781	TRIM33	-	OMIM	1p13.1	tripartite motif containing 33
114944063	114953561	LOC101928890	+			uncharacterized LOC101928890
115002456	115002802	RPL26P10	+		1p13.1	ribosomal protein L26 pseudogene 10
115010740	115012266	EIF2S2P5	+		1p13.1	eukaryotic translation initiation factor 2, subunit 2 beta pseudogene 5
115078592	115080077	PKMP1	+		1p13.2	pyruvate kinase, muscle pseudogene 1
115110178	115124265	BCAS2	-	OMIM	1p13.2	breast carcinoma amplified sequence 2
115127155	115212732	DENND2C	-		1p13.2	DENN/MADD domain containing 2C
115215719	115238239	AMPD1	-	OMIM	1p13	adenosine monophosphate deaminase 1
115247085	115259515	NRAS	-	OMIM	1p13.2	neuroblastoma RAS viral (v-ras) oncogene homolog
115259534	115300671	CSDE1	-	OMIM	1p22	cold shock domain containing E1, RNA-binding

start	stop	Symbol	O	Link	Cyto	Description
115312100	115323308	SIKE1	-	OMIM	1p13.2	suppressor of IKBKE 1
115377077	115394165	NR1H5P	+		1p13.2	nuclear receptor subfamily 1, group H, member 5, pseudogene
115397455	115537991	SYCP1	+	OMIM	1p13-p12	synaptonemal complex protein 1
115572445	115576930	TSHB	+	OMIM	1p13	thyroid stimulating hormone, beta
115590632	115632121	TSPAN2	-	OMIM	1p13.2	tetraspanin 2
