# Supplementary material for: 1p13.2 deletion displays clinical features overlapping Noonan syndrome, likely related to NRAS gene haploinsufficiency
Source: Genet Mol Biol. 2016 Aug 4;39(3):349–57. doi: 10.1590/1678-4685-GMB-2016-0049 (PMC5004838; doi:10.1590/1678-4685-GMB-2016-0049)
Supplement: Supplementary file 3 [file 1415-4757-gmb-1678-4685-GMB-2016-0049-Suppl03.pdf]

Table S3 - Deleted genes in our patient associated with Mendelian disease phenotypes in OMIM.

Gene name	Full gene name	Gene MIM ID	Inheritance	OMIM phenotype(s)
KCND3	Potassium voltage-gated channel, Shal-related subfamily, member 3	*605411	dom	Spinocerebellar ataxia 19, #607346
SLC16A1	Solute carrier family 16 (monocarboxylic acid transporters), member	*600682	dom	Erythrocyte lactate transporter defect, #245340; Hyperinsulinemic hypoglycemia, familial, 7, #610021
LRIG2	Leucine-Rich Repeats- and Immunoglobulin-like Domains-Containig Protein 2	*608869	rec	Urofacial syndrome 2, #615112
AP4B1	Adaptor-related protein complex 4, beta-1 subunit	*607245	rec	Spastic paraplegia 47, autosomal recessive, #614066
TRIM33	Tripartite motif-containing protein 33	*605769	dom	Thyroid carcinoma, papillary, #188550
AMPD1	Adenosine monophosphate deaminase-1, muscle	*102770	rec	Myopathy due to myoadenylate deaminase deficiency, #615511
NRAS	Neuroblastoma RAS viral (v-ras) oncogene homolog	*164790	dom	Colorectal cancer, #114500; Thyroid carcinoma, follicular, %188470; Autoimmune lymphoproliferative syndrome type IV, #614470; Noonan syndrome 6, #613224; Nevus, epidermal #162900
TSHB	Thyroid-stimulating hormone, beta polypeptide	*188540	rec	Hypothryoidism, congenital, nongoitrous 4, #275100
